# Propofol improves brain injury induced by chronic cerebral hypoperfusion in rats

**DOI:** 10.1002/fsn3.1915

**Published:** 2021-05-05

**Authors:** Xiaodong Wang, Xudong Yang, Fang Han, Ling Gao, Yi Zhou

**Affiliations:** ^1^ Department of Anesthesiology Peking University Hospital of Stomatology Beijing China

**Keywords:** chronic cerebral hypoperfusion, cognitive deficits, inflammatory response, oxidative stress, propofol

## Abstract

To study effect of propofol on cognitive dysfunction and brain injury in a rat model of chronic cerebral hypoperfusion. The bilateral carotid artery ligation (bilateral common carotid artery occlusion and BCCAO) to establish rat model of chronic cerebral hypoperfusion and randomly assigned to 4 groups (*n* = 10): sham‐operation group treated with saline model group, propofol treatment model group, normal saline treatment, propofol treatment in the sham‐operation group; continuous intraperitoneal injection of propofol and saline for 12 weeks. Morris water maze was used to evaluate the learning and memory ability of rats. Determination of central cholinergic and oxidative stress in brain tissue by spectrophotometry. Detection of inflammatory response in brain tissue by immunohistochemistry and ELISA method. Detection of neuronal loss in brain tissue by Nissl and TUNEL staining. Compared with the saline‐treated model group, propofol in model group significantly increased the rat brain tissue SOD activity (*p* < .01) and GPX activity (*p* < .01), decreased the MDA levels (*p* < .01) and protein carbonyl compound levels (*p* < .01). The propofol treatment of model group rats hippocampal GFAP‐immunoreactive satellite glial cells (*p* < .01) and immune Iba1‐positive microglia cells (*p* < .01) area percent compared to saline‐treated model group decreased significantly. The number of normal propofol treatment of model group rats hippocampus neuron than in physiological saline treatment model group rats was significantly increased (*p* < .01). Propofol can improve chronic cerebral hypoperfusion in rats induced by cognitive dysfunction and brain damage.

## Graphical abstract

In this work, we clearly evaluated propofol's effects in brain injury induced by chronic cerebral hypoperfusion treatment by vivo study.

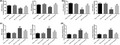

## 1 INTRODUCTION

Chronic cerebral hypoperfusion (CCH) is one of the main causes of the occurrence and development of mild cognitive impairment (MCI) (Kume et al., 2011; Staffen et al., 2006), vascular dementia (VD) (Gao et al., 2013; Schuff et al., 2009), and Alzheimer's disease (AD) (Gao et al., 2013; Nobili et al., 2009; Schuff et al., 2009). Since the pathophysiological mechanism of cognitive impairment and dementia caused by CCH is not clear, there is also a lack of specific clinical prevention and treatment measures (Baskys & Cheng, 2012; Levine & Langa, 2011). CCH‐induced brain injury, also known as chronic cerebral ischemia, is caused by a prolonged unchecked inadequacy of cerebral blood flow. A large number of studies have shown that (Hang et al., 2010) CCH‐induced brain injury can lead to the development of clinical cognitive impairment and VD. It was also found that bilateral common carotid arteries occlusion (BCCAO) in rat models can effectively simulate CCH injury (Zhao & Gong, 2015).

Propofol is a novel, rapid, and short‐acting intravenous anesthetic. In addition to hypnosis, sedation and amnesia, propofol also has the functions of reducing arterial blood pressure and inhibiting inflammatory response (Nie et al., 2016; Tang et al., 2014). In recent years, studies on the nonanesthetic effects of propofol have been focused on its anti‐inflammatory and antioxidant effects (Corcoran et al., 2011; Gong et al., 2016; Meng et al., 2013). Related studies have shown that propofol has antioxidant effects, and it can reduce intracellular calcium overload, inhibit apoptosis, alleviate neutrophils and endothelial cell adhesion, regulate the balance of inflammatory cytokines, and directly improve cell energy metabolism disorders (Tang et al., 2013; Tao et al., 2013). However, it is still unclear so far as to whether propofol can effectively improve the cognitive impairment and brain injury induced by CCH in rats.

In this study, the CCH rat models were established via bilateral common carotid arteries occlusion (Peng et al., 2007). Propofol was administered intraperitoneally for 12 weeks as a form of treatment, and multiple experimental techniques were used to explore the pharmacodynamic effect as well as explain the possible MOAs of propofol on cognitive impairment and brain injury in rats.

## 2 MATERIALS AND METHODS

### 2.1 Experimental animals and grouping

Forty adult male SD rats (6 months old, weight 380 ± 30 g) were purchased from the Experimental Animal Center of the Fourth Military Medical University. The rats were randomly divided into four groups (*n* = 10): saline treatment model group (BCCAO + Vehicle), propofol treatment model group (BCCAO + propofol), saline treatment sham‐operation group (Sham + Vehicle), and propofol treatment sham‐operation group (Sham + Propofol). The CCH rat models were established via bilateral common carotid arteries occlusion (BCCAO) (Peng et al., 2007): After inducing anesthesia, the bilateral common carotid arteries were separated by surgery and then occluded with 5‐0# suture; the rats in the sham‐operation group (sham‐operation) underwent the same operation procedure, but without that for BCCAO. Three days after the operation, the propofol (Sigma‐Aldrich) treatment group was given intra‐abdominal injection of propofol at a dosage of 5 mg/kg body weight (dissolved in 0.5 ml saline), while the placebo (vehicle) group was treated with the same dose of saline (0.5 ml), once a day, for 12 weeks. The rats were kept in a clean animal room, where they were given the freedom to eat and drink at any time.

### 2.2 Evaluation of spatial learning and memory function in rats

Morris water maze was used to evaluate the spatial learning and memory function of rats (Peng et al., 2007). The water maze was a circular pool (diameter = 120 cm, height = 50 cm, water depth = 31 cm) with water temperature regulated at 24 ± 1°C. It was divided into four quadrants, and a circular platform (diameter = 10 cm, height = 30 cm, or 1 cm below the water surface) lower than the water level was placed at the second quadrant 20 cm from the pool wall. During the experiment, the reference material around the pool remained unchanged. The experimental rats' movements were tracked and recorded in real time by an image acquisition system installed above the water maze. ①Evaluation of spatial learning ability: The rats were tested four times a day for a total of 5 days, within each of which the rats were placed into the water in different quadrants facing the pool wall to observe and record the time (latency) needed to climb up the platform. If the rat failed to find the platform within 120 s, it would be guided to the platform and kept there for 20 s, while the latency would be recorded as 120 s. The average latency after the tests was calculated. ②Spatial memory assessment: After 24 hr of the learning ability assessment, the second quadrant platform was removed, and the rats were placed into the water facing the pool wall in the four quadrants, and the time lengths needed by the rats to find the platform in the target quadrant (the second quadrant of the platform) were observed and recorded.

### 2.3 Preparation of brain tissues

At the end of behavioral experiment, the rats were anesthetized via intraperitoneal administration of pentobarbital sodium at a dose of 100 mg/kg body weight). The thoracic cavities were cut open, the hearts were exposed, the right auricles were cut open; the perfusion needles were inserted from the tip of the left ventricle, and 200 ml of icysaline was quickly dripped thereinto to wash the blood. The heads were decapitated and the brains taken out quickly, after which the left and right cerebral hemispheres were separated. The left cerebral cortex and hippocampus were quickly separated on ice and frozen in a refrigerator at −80°C; the right cerebral hemisphere was fixed with 4% paraformaldehyde, infiltrated with paraffin, and embedded into wax block 24 hr later.

### 2.4 Determination of oxidative stress index

After weighing the brain tissues, 9 parts by volume of cold saline (containing protease inhibitor) were added for homogenation and centrifuged at 4°C 3,000 *g* for 20 min, and the supernatant was taken and retained. The contents of SOD, GPX, MDA, and PC in brain tissues were determined by superoxide dismutase (SOD), glutathione peroxidase (GPX), malondialdehyde (MDA), and protein carbonyl compound (PC) kits (Nanjing Jiancheng Bioengineering Institute), respectively, according to the requirements of the kits (Ruan et al., 2010).

### 2.5 Detection of cholinergic markers

After weighing the brain tissues, 9 parts by volume of cold saline (containing protease inhibitor) were added for homogenation and centrifuged at 4°C 3,000 *g* for 20 min, and the supernatant was taken and retained. The contents of ACh, ChAT, and AChE in brain tissue were determined using acetylcholine (ACh), choline acetyl transferase (ChAT), and cholinesterase (AChE) kits (Nanjing Jiancheng Bioengineering Institute), respectively, according to the requirements of the kits (Ruan et al., 2010).

### 2.6 Detection of inflammatory cytokines

After weighing the brain tissues, the lysate containing protease inhibitor was added for homogenation and centrifuged at 4°C and 14,000 *g* for 25 min, before the supernatant was taken and retained. The contents of IL‐1 β, IL‐6, and TNF‐α in brain tissues were detected by interleukin‐1β (IL‐1β), interleukin‐6 (IL‐6), and tumor necrosis factor‐α (TNF‐α) ELISA kits (Invitrogen), according to the requirements of the kits (Ruan et al., 2010).

### 2.7 Detection of glial cells by immunohistochemical method

After paraffin‐infiltration followed by sectioning, dewaxing and hydration, the antigen retrieval was performed with protease K (0.2 mg/ml) and 10 mM sodium citrate solution (pH 6.0). At room temperature, 0.1% Triton X‐100 and 2% bovine serum albumin (BSA) were used to block nonspecific staining for 20 min. The sections were incubated with anti‐GFAP mouse monoclonal antibody (1:500 Millipore) and anti‐Iba1 rabbit monoclonal antibody (1:300 Wako Pure Chemical Industries, Ltd.) at 4°C for 24 hr, respectively; the sections were incubated with HRP‐labeled secondary antibody for 2 hr at room temperature and stained with diaminobenzidine (DAB) solution (Annaházi et al., 2007; Peng et al., 2007). Image‐Pro Plu (Media Cybernetics) was used to determine the area percentage of GFAP‐positive astrocyte and Iba1‐positive microglia in hippocampal area (Annaházi et al., 2007).

### 2.8 Nissl staining

After dewaxing and hydration, Nissl staining was performed with 0.5% cresyl violet solution for 1 min (Feng et al., 2012). The number of normal neurons in hippocampal CA1 area was quantitatively evaluated using Image‐Pro Plus (Media Cybernetics) (Feng et al., 2012).

### 2.9 TUNEL staining

The paraffin‐embedded sections of brain tissue were used for nuclear staining; in each group, 4–5 stained sections were selected; a microscope was used for observation, the apoptotic cells in visual field were recorded, and the apoptotic index of brain tissue was calculated.

### 2.10 Statistical processing

All data were expressed in mean ± standard error. SPSS15.0 was used for two‐way ANOVA and one‐way ANOVA, while Fisher's LSD was used to analyze the difference between groups. *p* < .05 was taken as the threshold for statistically significant difference.

## 3 RESULTS

### 3.1 Propofol alleviated the BCCAO‐induced learning and memory impairments

**FIGURE 1:**
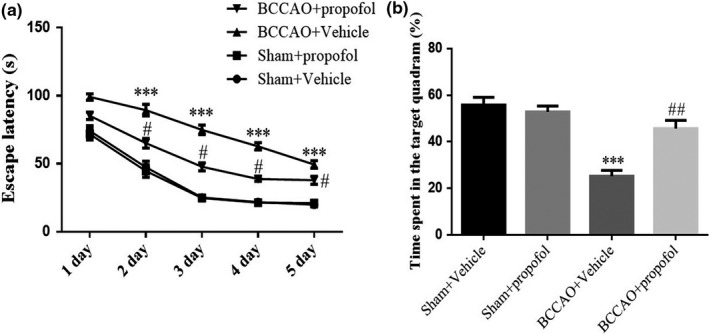
Effect of propofol on learning and memory impairments induced by BCCAO in rats. Sham + Vehicle: The sham rats were treated with vehicle; Sham + Prpofol: The sham rats were treated with propofol; BCCAO: The BCCAO rats were treated with vehicle; BCCAO + Propofol: The BCCAO rats were treated with propofol. ***: *p *< .001, compared with Sham‐Vehicle group; ##: *p *< .01, compared with BCCAO + Vehicle group

Figure 1a shows the time it took for the rats to find hidden platform (escape latency) during water maze learning and training. The statistical analysis shows that the escape latency of rats in the saline treatment model group, on the one hand, increased significantly compared with that of the sham‐operation group (*p* < .001), indicating that CCH causes significant learning impairment. On the other hand, the escape latency of rats in the propofol treatment model group decreased significantly compared with that of the saline treatment model group (*p* < .001), indicating that propofol can alleviate the BCCAO‐induced learning impairment inflicted to the rats.

Figure 1b shows the time needed for rats to find the platform in the target quadrant. The statistical analysis shows that the rats in the saline treatment model group, on the one hand, had significantly lesser time in the target quadrant than that in the sham‐operation group (*p* < .001), indicating that CCH causes significant memory impairment. On the other hand, the rats in the propofol treatment model group had significantly more time in the target quadrant than that in the saline treatment model group (*p* < .001), indicating that propofol can alleviate the BCCAO‐induced memory impairment inflicted to the rats.

### 3.2 Propofol recovered the BCCAO‐induced central cholinergic dysfunction

**FIGURE 2:**
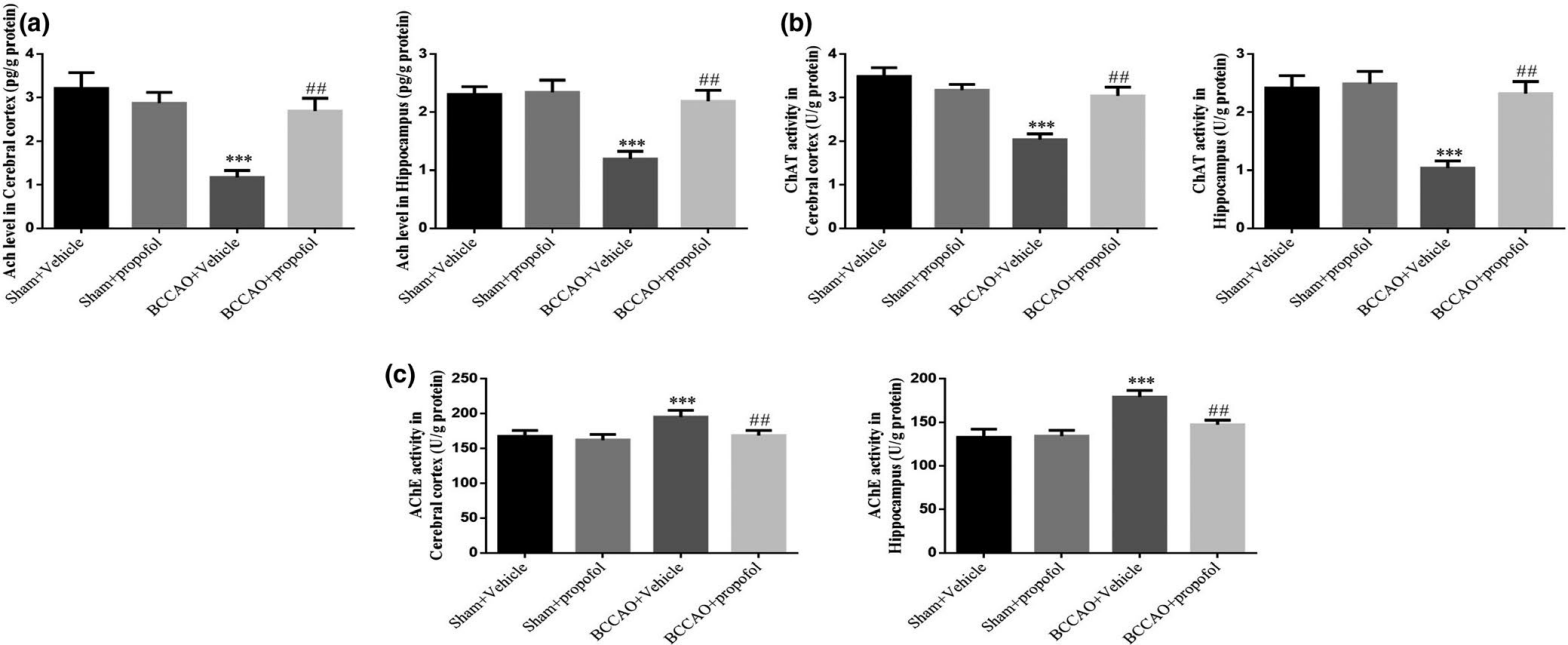
Effect of propofol on the central cholinergic dysfunction induced by BCCAO in rats. Sham + Vehicle: The sham rats were treated with vehicle; Sham + Prpofol: The sham rats were treated with propofol; BCCAO: The BCCAO rats were treated with vehicle; BCCAO + Propofol: The BCCAO rats were treated with propofol. ***: *p *< .001, compared with Sham‐Vehicle group; ##: *p *< .01, compared with BCCAO + Vehicle group

Compared with the sham‐operation group, the levels of ACh and the activities of ChAT (*p* < .001, respectively, Figure 2a,b) in the cerebral cortices and hippocampi of rats in the saline treatment model group significantly decreased, while the activity of AChE (*p* < .001, respectively, Figure 2c) increased significantly, indicating that CCH causes central cholinergic dysfunction in rats. Compared with the saline treatment model group, propofol significantly increased the ACh levels and ChAT activities (*p* < .01, respectively, Figure 2a,b) while also decreasing the AChE activities (*p* < .01, respectively, Figure 2c) in brain tissues of BCCAO rats after treatment, indicating that propofol can effectively repair CCH‐induced central cholinergic dysfunction.

### 3.3 Propofol improved the BCCAO‐induced oxidative stress responses

**FIGURE 3:**
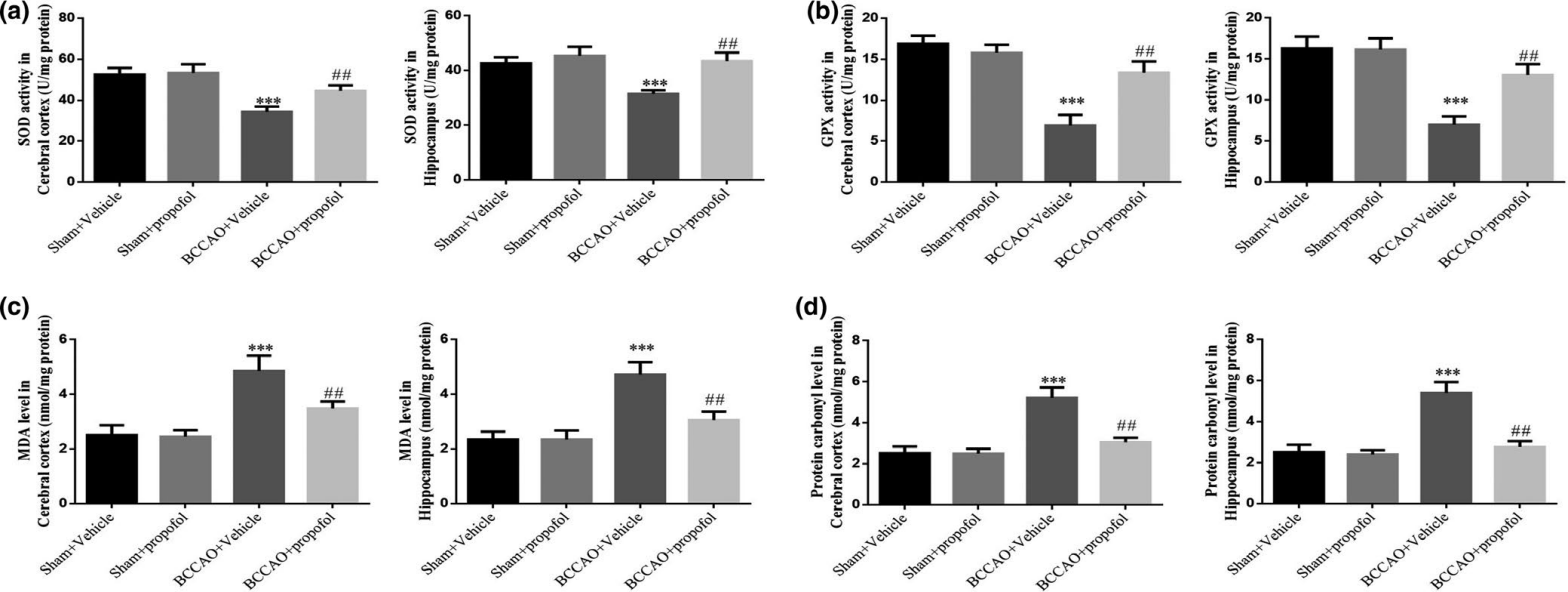
Effect of propofol on the oxidative stress induced by BCCAO in rats. Sham + Vehicle: The sham rats were treated with vehicle; Sham + Prpofol: The sham rats were treated with propofol; BCCAO: The BCCAO rats were treated with vehicle; BCCAO + Propofol: The BCCAO rats were treated with propofol. ***: *p *< .001, compared with Sham‐Vehicle group; ##: *p *< .01, compared with BCCAO + Vehicle group

Biochemical analysis results show that SOD activities and GPX activities (*p* < .001, respectively, Figure 3a,b) in cerebral cortices and hippocampi of rats in the saline treatment model group decreased significantly, while the MDA levels and protein carbonyl compound levels (*p* < .001, respectively, Figure 3c,d) increased significantly, compared with those in the sham‐operation group, indicating that CCH causes oxidative stress damage. Compared with the saline treatment model group, propofol significantly increased SOD activities and GPX activities (*p* < .01, respectively, Figure 3a,b), significantly decreased MDA levels and protein carbonyl compound levels (*p* < .01, respectively, Figure 3c,d) after the treatment, indicating that propofol can effectively ameliorate the effects of CCH‐induced oxidative stress damage.

### 3.4 Propofol inhibited the BCCAO‐induced inflammatory responses

**FIGURE 4:**
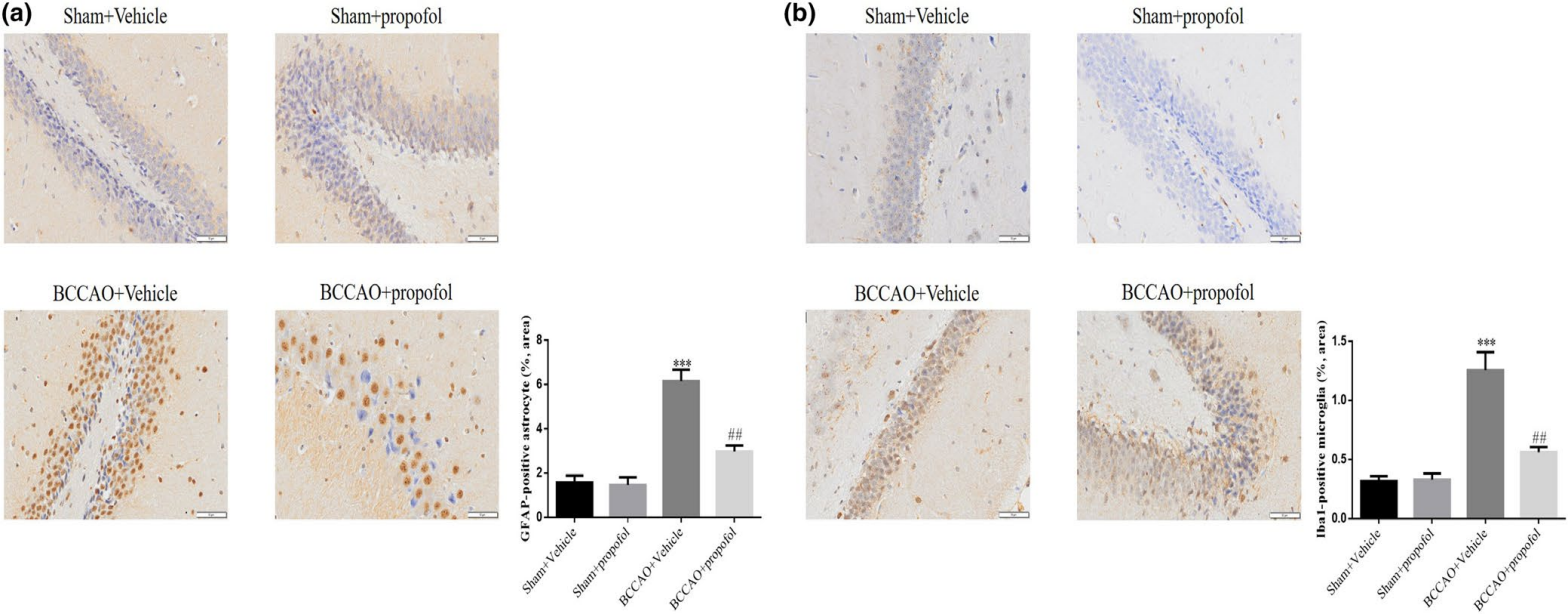
Propofol inhibited the BCCAO‐induced inflammatory responses. Sham + Vehicle: The sham rats were treated with vehicle; Sham + Prpofol: The sham rats were treated with propofol; BCCAO: The BCCAO rats were treated with vehicle; BCCAO + Propofol: The BCCAO rats were treated with propofol. (a) GFAP‐positive astrocyte in difference groups. ***: *p *< .001, compared with Sham‐Vehicle group; ##: *p *< .01, compared with BCCAO + Vehicle group. (b) Iba1‐positive microglia in difference groups. ***: *p *< .001, compared with Sham‐Vehicle group; ##: *p *< .01, compared with BCCAO + Vehicle group

**FIGURE 5:**
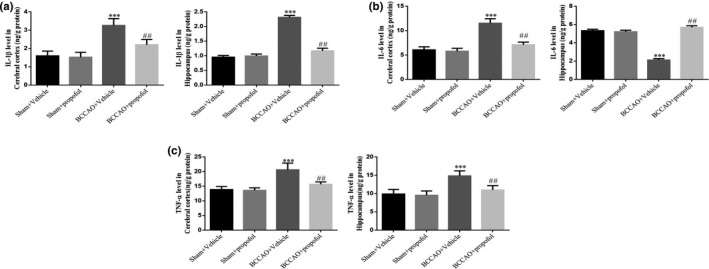
Effect of prpofol on proinflammatory cytokines induced by BCCAO in rats. Sham + Vehicle: The sham rats were treated with vehicle; Sham + Prpofol: The sham rats were treated with propofol; BCCAO: The BCCAO rats were treated with vehicle; BCCAO + Propofol: The BCCAO rats were treated with propofol. ***: *p *< .001, compared with Sham‐Vehicle group; ##: *p *< .01, compared with BCCAO + Vehicle group

The immunohistochemistry results show that, compared with the sham‐operation group, a large number of GFAP‐immunoreactive astrocytes (Figure 4a) and Iba1‐immunoreactive microglia (Figure 4b) were found in the hippocampi of the rats in the saline treatment model group; the number of GFAP‐immunoreactive astrocytes (Figure 4a) and Iba1‐immunoreactive microglia (Figure 4b) in the hippocampus of the rats in the propofol treatment model group decreased significantly. However, no significantly activated glial cells were observed in the cerebral cortex of rats in each group. A quantitative statistical analysis shows that the area percentage of GFAP‐immunoreactive astrocytes (*p* < .001, Figure 4a) and Iba1‐immunoreactive microglia (*p* < .001, Figure 4b) in the hippocampus of the rats in the saline treatment model group increased significantly, compared with that of the sham‐operation group; while the area percentage of GFAP‐immunoreactive astrocytes (*p* < .01, Figure 4a) and Iba1‐immunoreactive microglia (*p* < .01, Figure 4b) in the hippocampus of the rats in the propofol treatment model group decreased significantly, compared with that of saline treatment model group. The study results showed that CCH could lead to the activation of glial cells, while propofol could effectively inhibit the activation of glial cells (Figure 5).

**FIGURE 6:**
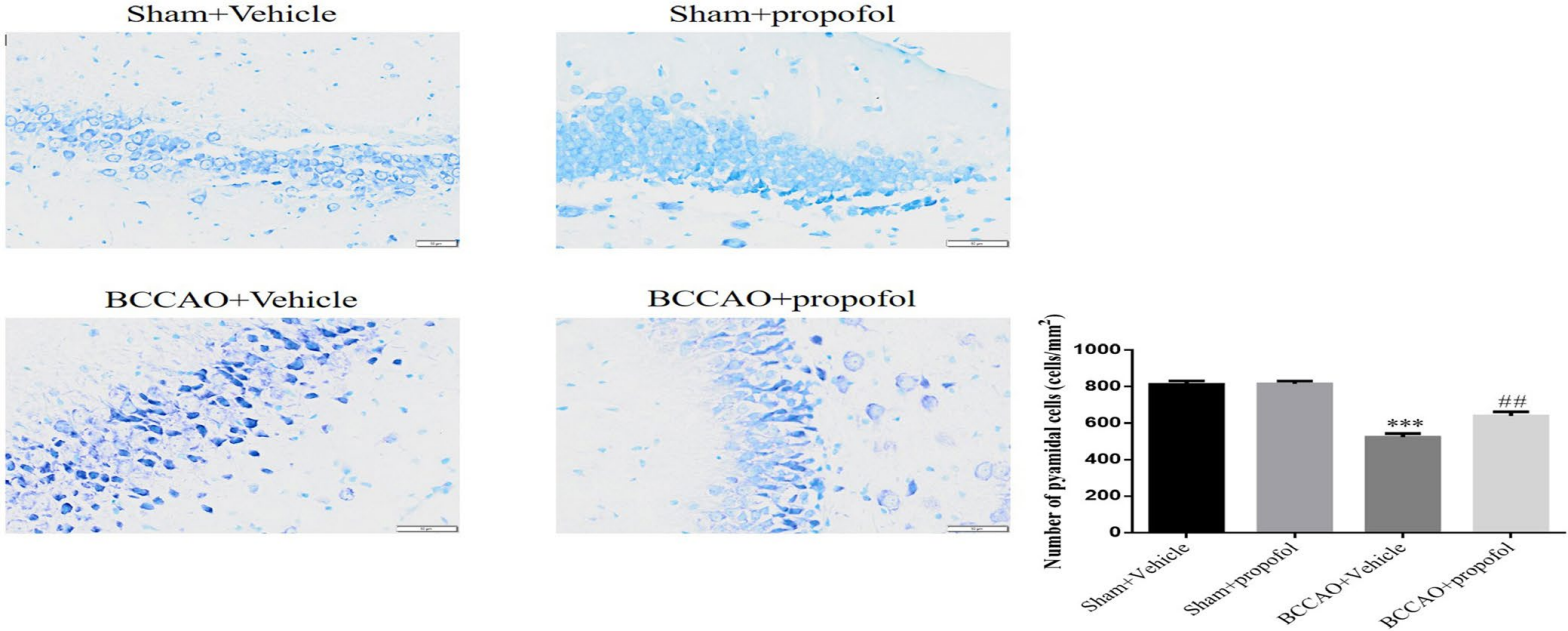
Effect of prpofol on the neuronal damage induced by BCCAO in the hippocampus of rats. Sham + Vehicle: The sham rats were treated with vehicle; Sham + Prpofol: The sham rats were treated with propofol; BCCAO: The BCCAO rats were treated with vehicle; BCCAO + Propofol: The BCCAO rats were treated with propofol. ***: *p *< .001, compared with Sham‐Vehicle group; ##: *p *< .01, compared with BCCAO + Vehicle group

The results of ELISA analysis show that the levels of IL‐1 β (*p* < .001, respectively, Figure 6a), IL‐6 (*p* < .001, respectively, Figure 6b) and TNF‐α (*p* < .001, respectively, Figure 6c) in the brain tissues of rats in the saline treatment model group increased significantly, compared with those in the sham‐operation group; while propofol significantly reduced the levels of IL‐1 β (*p* < .01, respectively, Figure 6a), IL‐6 (*p* < .01, respectively, Figure 6b) and TNF‐α (*p* < .01, respectively, Figure 6c) in the brain tissues of BCCAO rats. These results suggested that CCH can increase the release of inflammatory cytokines, which propofol can effectively reduce.

### 3.5 Propofol reduced neuronal injury in BCCAO rats

Nissl staining shows the absence of injured neurons in the hippocampi of rats in the sham‐operation group (Figure 6), whereas a large number of injured neurons were found in the hippocampi of rats in the saline treatment model group (Figure 6), and a small number of injured neurons were found in the hippocampi of rats in the propofol treatment model group (Figure 6). However, no injured neurons were observed in the cerebral cortices of rats in each group. The quantitative analysis shows that the number of normal neurons in the hippocampi of rats in the saline treatment model decreased significantly, compared with that of the sham‐operation group (*p* < .001, Figure 6), while the number of normal neurons in the hippocampi of rats in the propofol treatment model group increased significantly, compared with that of the saline treatment model group (*p* < .01, Figure 6).

### 3.6 Propofol ameliorated apoptosis in brain tissues of BCCAO rats

**FIGURE 7:**
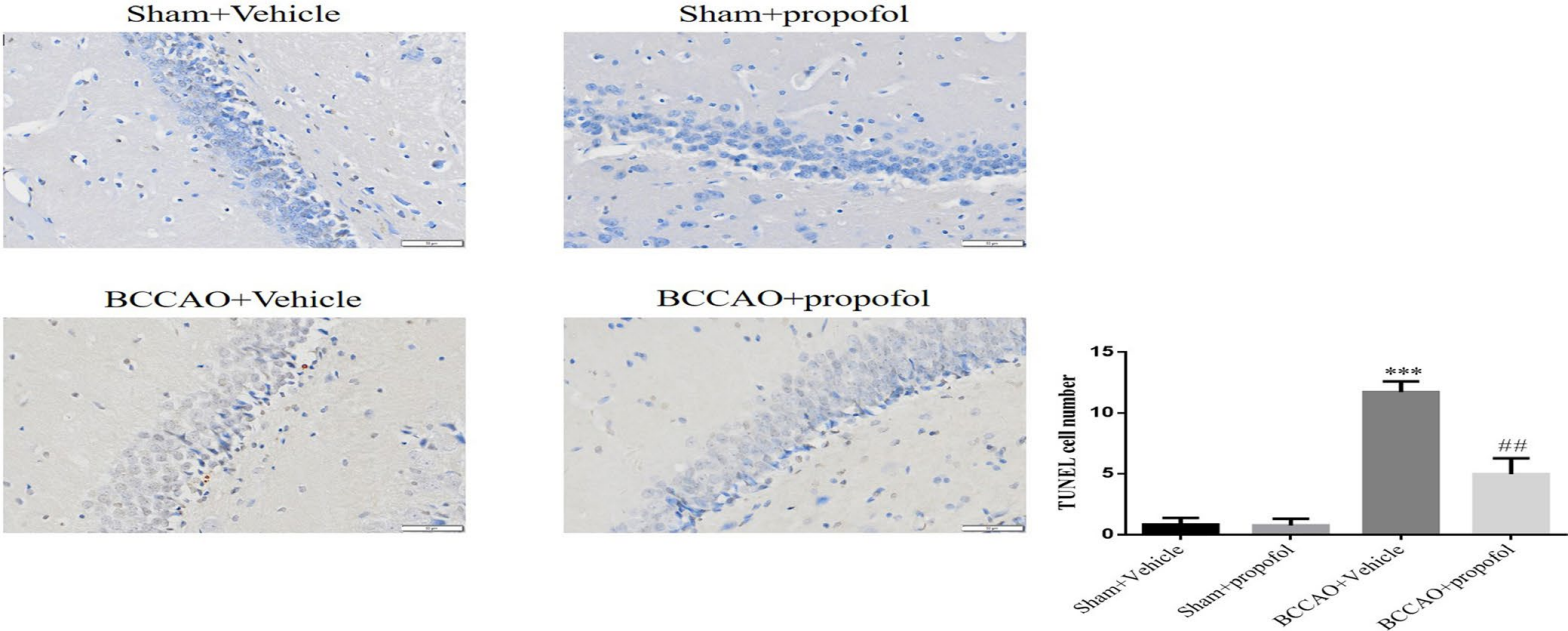
Propofol ameliorated apoptosis in brain tissues of BCCAO rats. Sham + Vehicle: The sham rats were treated with vehicle; Sham + Prpofol: The sham rats were treated with propofol; BCCAO: The BCCAO rats were treated with vehicle; BCCAO + Propofol: The BCCAO rats were treated with propofol. ***: *p *< .001, compared with Sham‐Vehicle group; ##: *p *< .01, compared with BCCAO + Vehicle group

TUNEL staining show that a small number of apoptotic cells were found in the hippocampi of rats in the sham‐operation group (Figure 7), a large number of apoptotic cells in the hippocampi of rats in the saline treatment model group (Figure 7), and a small number of apoptotic cells in the hippocampi of rats in the propofol treatment model group (Figure 7). The quantitative analysis shows that the number of apoptotic cells in the hippocampi of rats in the saline treatment model increased significantly, compared with that of the sham‐operation group (*p* < .001, Figure 7), while the number of apoptotic cells in the hippocampi of rats in the propofol treatment model group decreased significantly, compared with that of the saline treatment model group (*p* < .01, Figure 7).

## 4 DISCUSSION

Studies have shown that CCH can lead to spatial learning and memory impairments (Cechetti et al., 2012; Li et al., 2012; Vicente et al., 2009) as well as nonspatial memory impairment (Sarti et al., 2002). The results acquired herein are consistent with these studies: The Morris water maze test shows that BCCAO rats in the placebo group had obvious spatial learning and memory impairment, while propofol could effectively mend the spatial learning and memory impairments induced by BCCAO in rats. Therefore, this study speculates that the effect of propofol on cognitive impairment in rats may be achieved by reducing the biochemical and neuropathological changes caused by CCH in the brain.

Although CCH can lead to cognitive impairment in rats, the exact mechanism has not been fully clarified (Farkas et al., 2007). Oxidative stress caused by the imbalance between free radicals and antioxidant system is recognized to be involved in the pathological process of cognitive impairment, especially in diseases such as VD and AD (). On the one hand, this study found that antioxidant enzymes (SOD and GPX) in the brain of BCCAO rats in the placebo group decreased significantly, compared with those in the sham‐operation group, whereas lipid oxide (MDA) and protein oxide (carbonyl compound) increased significantly, indicating that CCH causes substantial oxidative stress injury. On the other hand, the propofol treatment effectively ameliorated the abnormal changes of oxidative stress injury markers in the brain of BCCAO rats, suggesting that the effects of propofol on cognitive impairment in rats are directly related to its antioxidant activity.

It is well known that the central cholinergic system plays an important role in the formation of learning and memory (Farkas et al., 2007). Studies have shown that CCH decrease ChAT activities and ACh levels in rats' brains (Choi et al., 2011; Kumaran et al., 2008; Tanaka et al., 1996), and that it is closely related to cognitive impairment in rats (Tanaka et al., 1996). The findings herein show that the activities of ChAT and ACh in the rats' brains in the saline treatment model group decreased significantly, compared with those of the sham‐operation group, while the activities of AChE increased significantly, which further prove that CCH can lead to the disorder of the central cholinergic system (Choi et al., 2011; Kumaran et al., 2008; Tanaka et al., 1996). However, the administration of propofol significantly increased ChAT activities and ACh levels, while also decreasing AChE activities in the brain tissues of BCCAO rats. Previous studies have shown ChAT activity and ACh level in brain to be closely related to cognitive impairment in rats (Tanaka et al., 1996), while the findings herein show that propofol can ameliorate central cholinergic dysfunction and contribute to the improvement of learning capability and memory capacity of BCCAO rats.

Previous experiments have proven that inflammatory cytokines are mainly produced and released by vascular endothelial cells, glial cells, and neurons, all of which are important triggers of brain inflammatory response and brain injury (Peng et al., 2007; Vicente et al., 2009). CCH can lead to the activation of glial cells and the excessive release of inflammatory cytokines, resulting in neuronal injury (Cechetti et al., 2012; Peng et al., 2007; Vicente et al., 2009; Zhang et al., 2011). In this study, it was found that the numbers of the activated astrocytes and microglia as well as the levels of inflammatory cytokines (IL‐1 β, IL‐6, and TNF‐α) in the hippocampi of rats in the saline treatment model group increased significantly; while propofol significantly reduced the inflammatory response in the brain of BCCAO rats, suggesting that propofol can not only inhibit the activation of glial cells, but also reduce the release of inflammatory cytokines.

Many studies have shown that CCH can cause selective injury or loss of neurons in the susceptible areas of the brain, especially in the hippocampal CA1 area (Annaházi et al., 2007; Peng et al., 2007). The histopathological results of this study showed that there were significant neuronal injuries and apoptosis in hippocampal CA1 area of the rats in saline treatment model group, while the propofol treatment has significantly reduced the CCH‐induced neuronal injury. The research results herein are consistent with those observed in previous studies, that is, propofol can effectively protect rat neurons from neuronal injury, loss and apoptosis induced by focal cerebral ischemia (Corcoran et al., 2011; Khan et al., 2009; Rodrigues et al., 2013). Since learning and memory function depends on the integrity of hippocampal structure (Li et al., 2011), the protective effect of propofol on hippocampal neurons may play an important role in ameliorating CCH‐induced cognitive impairment.

In conclusion, this study firstly proves that propofol acts on multiple pathophysiological processes in the brain through multi‐target pharmacology, thus effectively ameliorating cognitive dysfunction and brain injury caused by CCH. Our results show that propofol can potentially emerge as a new candidate drug that can be used to treat cognitive impairments and brain damages related to CCH, hence providing an experimental basis for the development of new drugs for the prevention and treatment of VD and AD.

## CONFLICT OF INTEREST

None.

## STUDIES INVOLVING ANIMAL OR HUMAN SUBJECTS

This study was approved by ethics committee of Peking University Hospital of Stomatology.

## References

[fsn31915-bib-0001] Annaházi, A. , Mracskó, E. , Süle, Z. , Karg, E. , Penke, B. , Bari, F. , & Farkas, E. (2007). Pre‐treatment and post‐treatment with alpha‐tocopherol attenuates hippocampal neuronal damage in experimental cerebral hypoperfusion. European Journal of Pharmacology, 571(2–3), 120–128. 17597609 10.1016/j.ejphar.2007.05.048

[fsn31915-bib-0002] Baskys, A. , & Cheng, J.X. (2012). Pharmacological prevention and treatment of vascular dementia: Approaches and perspectives. Experimental Gerontology, 47(11), 887–891. 10.1016/j.exger.2012.07.002 22796225

[fsn31915-bib-0003] Cechetti, F. , Pagnussat, A.S. , Worm, P.V. , Elsner, V.R. , Ben, J. , da Costa, M.S. , Mestriner, R. , Weis, S.N. , & Netto, C.A. (2012). Chronic brain hypoperfusio causes early glial activation and neuronal death, and subsequent long‐term memory impairment. Brain Research Bulletin, 87(1), 109–116. 22040859 10.1016/j.brainresbull.2011.10.006

[fsn31915-bib-0004] Choi, B.R. , Kwon, K.J. , Park, S.H. , Jeon, W.K. , Han, S.‐H. , Kim, H.Y. , & Han, J.‐S. (2011). Alternations of septal‐hippocampal system in the adult wistar rat with spatial memory impairments induced by chronic cerebral hypoperfusion. Experimental Neurobiology, 20(2), 92–99. 10.5607/en.2011.20.2.92 22110366 PMC3213701

[fsn31915-bib-0005] Corcoran, T.B. , Barden, A.E. , Mas, E. , Grape, S. , Koren, V. , Phillips, M. , Roberts, L.J. , & Mori, T.A. (2011). Hemoglobin attenuates the effects of inspired oxygen on plasma isofurans in humans during upper‐limb surgery. Free Radical Biology and Medicine, 51(6), 1235–1239. 10.1016/j.freeradbiomed.2011.06.026 21763419 PMC3157081

[fsn31915-bib-0006] Farkas, E. , Luiten, P.G. , & Bari, F. (2007). Permanent, bilateral common carotid artery occlusion in the rat: A model for chronic cerebral hypoperfusion‐related neurodegenerative diseases. Brain Research Reviews, 54(1), 162–180. 10.1016/j.brainresrev.2007.01.003 17296232

[fsn31915-bib-0007] Feng, Z. , Lu, Y. , Wu, X. , Zhao, P. , Li, J. , Peng, B. , Qian, Z. , & Zhu, L. (2012). Ligustilide alleviates brain damage and improves cognitive function in rats of chronic cerebral hypoperfusion. Journal of Ethnopharmacology, 144(2), 313–321. 10.1016/j.jep.2012.09.014 23022689

[fsn31915-bib-0008] Gao, Y.Z. , Zhang, J.J. , Liu, H. , Wu, G.‐Y. , Xiong, L. , & Shu, M. (2013). Regional cerebral blood flow and cerebrovascular reactivity in Alzheimer's disease and vascular dementia assessed by arterial spinlabeling magnetic resonance imaging. Current Neurovascular Research, 10(1), 49–53. 10.2174/156720213804806016 23151075

[fsn31915-bib-0009] Gong, H.Y. , Zheng, F. , Zhang, C. , Chen, X.‐Y. , Liu, J.‐J. , & Yue, X.‐Q. (2016). Propofol protects hippocampal neurons from apoptosis in ischemic brain injury by increasing GLT‐1 expression and inhibiting the activation of NMDAR via the JNK/Akt signaling pathway. International Journal of Molecular Medicine, 38(3), 943–950. 10.3892/ijmm.2016.2663 27430327

[fsn31915-bib-0010] Hang, H. , Sato, T. , Hirao, K. , Kanetaka, H. , Iwamoto, T. , & Koizumi, K. (2010). The progression of cognitive deterioration and regional cerebral blood flow patterns in Alzheimer's disease: A longitudinal SPECT study. Journal of the Neurological Sciences, 290(1–2), 96–101. 10.1016/j.jns.2009.10.022 19931870

[fsn31915-bib-0011] Khan, M.M. , Ahmad, A. , Ishrat, T. , Khuwaja, G. , Srivastawa, P. , Khan, M.B. , Raza, S.S. , Javed, H. , Vaibhav, K. , Khan, A. , & Islam, F. (2009). Rutin protects the neural damage induced by transient focal ischemia in rats. Brain Research, 1292, 123–135. 10.1016/j.brainres.2009.07.026 19631195

[fsn31915-bib-0012] Kumaran, D. , Udayabanu, M. , Kumar, M. , Aneja, R. , & Katyal, A. (2008). Involvement of angiotensin converting enzyme in cerebral hypoperfusion induced anterograde memory impairment and cholinergic dysfunction in rats. Neuroscience, 155(3), 626–639. 10.1016/j.neuroscience.2008.06.023 18621107

[fsn31915-bib-0013] Kume, K. , Hanyu, H. , Sato, T. , Hirao, K. , Shimizu, S. , Kanetaka, H. , Sakurai, H. , & Iwamoto, T. (2011). Vascular risk factors are associated with faster decline of Alzheimer disease: A longitudinal SPECT study. Journal of Neurology, 258(7), 1295–1303. 10.1007/s00415-011-5927-y 21327852

[fsn31915-bib-0014] Levine, D.A. , & Langa, K.M. (2011). Vascular cognitive impairment: Disease mechanisms and therapeutic implications. Neurotherapeutics: the Journal of the American Society for Experimental NeuroTherapeutics, 8(3), 361–373. 10.1007/s13311-011-0047-z 21556678 PMC3167237

[fsn31915-bib-0015] Li, E. , Kim, D.H. , Cai, M. , Lee, S. , Kim, Y. , Lim, E. , Hoon Ryu, J. , G Unterman, T. , & Park, S. (2011). Hippocampus‐dependent spatial learning and memory are impaired in growth hormone‐deficient spontaneous dwarf rats. Endocrine Journal, 58(4), 257–267. 10.1507/endocrj.K11E-006 21350302

[fsn31915-bib-0016] Li, Y. , He, Y. , Guan, Q. , Liu, W. , Han, H. , & Nie, Z. (2012). Disrupted iron metabolism and ensuing oxidative stress maymediate cognitive dysfunction induced by chronic cerebral hypoperfusion. Biological Trace Element Research, 150(1–3), 242–248. 10.1007/s12011-012-9455-0 22639386

[fsn31915-bib-0017] Meng, T. , Yu, J. , Lei, Z. , Wu, J. , Wang, S. , Bo, Q. , Zhang, X. , Ma, Z. , & Yu, J. (2013). Propofol reduces lipopolysaccharide induced, NADPH oxidase (NOX 2) mediated TNF‐alpha and IL‐6 production in macrophages. Clinical and Developmental Immunology, 2013, 325481. 24371447 10.1155/2013/325481 PMC3859231

[fsn31915-bib-0018] Nie, Y. , Song, R. , Chen, W. , Qin, Z. , Zhang, J. , & Tang, J. (2016). Effects of stellate ganglion block on cerebrovascular vasodilation in elderly patients and patients with subarachnoid haemorrhage. British Journal of Anaesthesia, 117(1), 131–132. 10.1093/bja/aew157 27317713 PMC4913408

[fsn31915-bib-0019] Nobili, F. , De Carli, F. , Frisoni, G.B. , Portet, F. , Verhey, F. , Rodriguez, G. , Caroli, A. , Touchon, J. , Morbelli, S. , Guerra, U.P. , Dessi, B. , Brugnolo, A. , & Visser, P.J. (2009). SPECT predictors of cognitive decline and Alzheimer's disease in mild cognitive impairment. Journal of Alzheimer's Disease, 17(4), 761–772. 10.3233/JAD-2009-1091 19542623

[fsn31915-bib-0020] Peng, Y. , Xu, S. , Chen, G. , Wang, L. , Feng, Y. , & Wang, X. (2007). l‐3‐n‐Butylphthalide improves cognitive impairment induced by chronic cerebral hypoperfusion in rats. Journal of Pharmacology and Experimental Therapeutics, 321(3), 902–910. 10.1124/jpet.106.118760 17374747

[fsn31915-bib-0021] Rodrigues, A.M. , Marcilio Fdos, S. , Frazão Muzitano, M. , & Giraldi‐Guimarães, A. (2013). Therapeutic potential of treatment with the flavonoid rutin after cortical focal ischemia in rats. Brain Research, 1503, 53–61. 10.1016/j.brainres.2013.01.039 23370003

[fsn31915-bib-0022] Ruan, C.J. , Li, Z. , Zhang, L. , Chen, D.H. , Du, G.H. , & Sun, L. (2010). Protective effects of trans‐2, 4‐dimethoxystibene on cognitive, impairments induced by Abeta (25–35) in, hypercholesterolemic rats. Brain Research Bulletin, 82(5–6), 251–258. 20451590 10.1016/j.brainresbull.2010.04.016

[fsn31915-bib-0023] Sarti, C. , Pantoni, L. , Bartolini, L. , & Inzitari, D. (2002). Persistent impairment of gait performances and working memory after bilateral common carotid artery occlusion in the adult Wistar rat. Behavioral Brain Research, 136(1), 13–20. 10.1016/S0166-4328(02)00090-6 12385786

[fsn31915-bib-0024] Schuff, N. , Matsumoto, S. , Kmiecik, J. , Studholme, C. , Du, A. , Ezekiel, F. , Miller, B.L. , Kramer, J.H. , Jagust, W.J. , Chui, H.C. , & Weiner, M.W. (2009). Cerebral blood flow in ischemic vascular dementia and Alzheimer's disease, measured by arterial spin‐labeling magnetic resonance imaging. Alzheimer's & Dementia: the Journal of the Alzheimer's Association, 5(6), 454–462. 10.1016/j.jalz.2009.04.1233 PMC2802181 19896584

[fsn31915-bib-0025] Staffen, W. , Schonauer, U. , Zauner, H. , Spindler, I. , Mair, A. , Iglseder, B. , Bernroider, G. , & Ladurner, G. (2006). Brain perfusion SPECT in patients with mild cognitive impairment and Alzheimer's disease: Comparison of a semiquantitative and a visual evaluation. Journal of Neural Transmission (Vienna), 113(2), 195–203. 10.1007/s00702-005-0321-5 15959843

[fsn31915-bib-0026] Tanaka, K. , Ogawa, N. , Asanuma, M. , Kondo, Y. , & Nomura, M. (1996). Relationship between cholinergic dysfunction and discrimination learning disabilities in Wistar rats following chronic cerebral hypoperfusion. Brain Research, 729(1), 55–65. 10.1016/0006-8993(96)00400-3 8874876

[fsn31915-bib-0027] Tang, J. , Hu, J.J. , Lu, C. , Liang, J.‐N. , Xiao, J.‐F. , Liu, Y.‐T. , Lin, C.‐S. , & Qin, Z.‐S. (2014). Propofol inhibits lipopolysaccharide‐induced tumor necrosis factor‐alpha expression and myocardial depression through decreasing the generation of superoxide anion in cardiomyocytes. Oxidative Medicine and Cellular Longevity, 2014, 1–12. 10.1155/2014/157376 PMC4144395 25180066

[fsn31915-bib-0028] Tang, J. , Jiang, Y. , Tang, Y. , Chen, B. , Sun, X. , Su, L. , & Liu, Z. (2013). Effects of propofol on damage of rat intestinal epithelial cells induced by heat stress and lipopolysaccharides. Brazilian Journal of Medical and Biological Research, 46(6), 507–512. 10.1590/1414-431X20132785 23802227 PMC3854439

[fsn31915-bib-0029] Tao, T. , Zhao, Z. , Hao, L. , Gu, M. , Chen, L. , & Tang, J. (2013). Propofol promotes proliferation of cultured adult rat hippocampal neural stem cells. Journal of Neurosurgical Anesthesiology, 25(3), 299–305. 10.1097/ANA.0b013e31828baa93 23519370

[fsn31915-bib-0030] Vicente, E. , Degerone, D. , Bohn, L. , Scornavaca, F. , Pimentel, A. , Leite, M.C. , Swarowsky, A. , Rodrigues, L. , Nardin, P. , Vieira de Almeida, L.M. , Gottfried, C. , Souza, D.O. , Netto, C.A. , & Gonçalves, C.A. (2009). Astroglial and cognitive effects of chronic cerebral hypoperfusion in the rat. Brain Research, 1251, 204–212. 10.1016/j.brainres.2008.11.032 19056357

[fsn31915-bib-0031] Zhang, G.L. , Deng, J.P. , Wang, B.H. , Zhao, Z.‐W. , Li, J. , Gao, L. , Liu, B.‐L. , Xong, J.‐R. , Guo, X.‐D. , Yan, Z.‐Q. , & Gao, G.‐D. (2011). Gypenosides improve cognitive impairment induced by chronic cerebral hypoperfusion in rats by suppressing oxidative stress and astrocytic activation. Behavioural Pharmacology, 22(7), 633–644. 10.1097/FBP.0b013e32834afef9 21897202

[fsn31915-bib-0032] Zhao, Y. , & Gong, C.X. (2015). From chronic cerebral hypoperfusion to Alzheimer‐like brain pathology and neurodegeneration. Cellular and Molecular Neurobiology, 35(1), 101–110. 10.1007/s10571-014-0127-9 25352419 PMC11486181

